# Biallelic *CACNA1A* variants: Review of literature and report of a child with drug‐resistant epilepsy and developmental delay

**DOI:** 10.1002/ajmg.a.62960

**Published:** 2022-09-05

**Authors:** Vivien M. Y. Wong‐Spracklen, Anna Kolesnik, Josefine Eck, Saras Sabanathan, Olivera Spasic‐Boskovic, Anna Maw, Kate Baker

**Affiliations:** ^1^ Department of Paediatric Neurology Cambridge University Hospitals NHS Foundation Trust Cambridge UK; ^2^ MRC Cognition and Brain Sciences Unit University of Cambridge Cambridge UK; ^3^ Department of Paediatric Neurosciences Evelina Childrens Hospital London UK; ^4^ East Genomic Laboratory Hub Cambridge University Hospitals NHS Foundation Trust Cambridge UK; ^5^ Department of Medical Genetics University of Cambridge Cambridge UK

**Keywords:** biallelic, CACNA1A, cerebellar atrophy, epileptic encephalopathy, intellectual disability, recessive

## Abstract

Biallelic variants in *CACNA1A* have previously been reported in nine individuals (four families) presenting with epilepsy and cognitive impairments of variable severity and age‐of‐onset. Here, we describe a child who presented at 6 months of age with drug‐resistant epilepsy and developmental delay. At 10 years of age, she has profound impairments in motor function and communication. MRI was initially unremarkable, but progressed to severe cerebellar atrophy by age 3 years. Next Generation Sequencing and panel analysis identified a maternally inherited truncating variant c.2042_2043delAG, p.(Gln681ArgfsTer100) and paternally inherited missense variant c.1693G>A, p.(Glu565Lys). In contrast to previously reported biallelic cases, parents carrying these monoallelic variants did not display clear signs of a *CACNA1A*‐associated syndrome. In conclusion, we provide further evidence that biallelic *CACNA1A* variants can cause a severe epileptic and developmental encephalopathy with progressive cerebellar atrophy, and highlight complexities of genetic counseling in such situations.

## 1 INTRODUCTION

*CACNA1A* (OMIM *601011) encodes the alpha‐1a subunit of the voltage‐dependent P/Q calcium channel. The channel is widely expressed throughout the central nervous system and regulates multiple calcium‐dependent processes including neuronal excitability and pre‐synaptic control of vesicle release (Mochida, 2018). Pathogenic variants in *CACNA1A* were initially associated with autosomal dominant neurological disorders characterized by paroxysmal or progressive motor impairments. Truncating variants typically cause episodic ataxia type 2 (EA2 OMIM #108500), missense variants are associated with familial hemiplegic migraine, with or without ataxia (OMIM #141500), while CAG triplet repeat expansions cause spinocerebellar ataxia type 6 (OMIM #183086).

More recently, the phenotypic spectrum associated with inherited or de novo heterozygous *CACNA1A* variants has broadened to encompass epilepsies and cognitive impairments (Damaj et al., 2015; Epi4K‐Consortium, 2016). This phenotype has been classified as developmental and epileptic encephalopathy type 42 (DEE 42; OMIM #617106). Seizure phenotypes are complex, and can be early onset and refractory (Le Roux et al., 2021). Paroxysmal non‐epileptic abnormalities of movement and oculomotor function are also reported (Gur‐Hartman et al., 2021). Cognitive impairments range from mild learning difficulties to moderate intellectual disability (ID), irrespective of the type and severity of episodic motor symptoms, correlating with cerebellar atrophy on MRI (Humbertclaude et al., 2020). There is no clear evidence that epilepsies and ID reflect gain‐of‐function rather than loss‐of‐function molecular mechanisms, and no clear genotype–phenotype correlations have been observed (Gur‐Hartman et al., 2021; Jiang et al., 2019; Niu et al., 2021).

A general principle in childhood neurogenetic disorders is that, rarely, individuals are diagnosed with biallelic variants in a gene typically associated with heterozygous variants. If both variants are pathogenic, severe early‐onset clinical presentation resembling the dominant disorder is predicted. This has been observed for several disorders with similar clinical characteristics and presynaptic mechanisms to *CACNA1A*, including *CACNA1B* (Gorman et al., 2019), *STXBP1* (Lammertse et al., 2020) and *PRRT2* (Ebrahimi‐Fakhari et al., 2015). Previously reported *CACNA1A* biallelic cases are summarized in Table S1. Compound heterozygous variants were first reported in two siblings with DEE plus progressive cerebral, cerebellar, and optic nerve atrophy (Reinson et al., 2016). Family members with heterozygous variants manifested ID and ataxia. A similar patient has since been reported (Ko et al., 2021). Recently, a *CACNA1A* homozygous truncating variant (p.Arg932*) was reported in a consanguineous family. Four children with these biallelic variants presented with DEE, leading to death by 6 months of age, whilst both carrier parents had symptoms consistent with EA2 (Arteche‐Lopez et al., 2021). Finally, and more speculatively, a family has been reported in which an insertion/deletion in exon 47, predicted to be deleterious to protein function, is associated with adult‐onset progressive myoclonic epilepsy, cognitive decline and ataxia (Lv et al., 2017).

Here, we report a fifth family in which biallelic *CACNA1A* variants are associated with a severe neurodevelopmental phenotype. However, family members with heterozygous *CACNA1A* variants are either asymptomatic or have mild and non‐specific features. Reduced penetrance and high degrees of intra‐familial variability have been reported across all *CACNA1A* phenotypes. This family emphasizes the increasing complexity of variant interpretation and genetic counseling in relation to this gene (Angelini et al., 2019; Hommersom et al., 2021).

## 2 CLINICAL REPORT

### 2.1 Medical and neurological history

**FIGURE 1:**
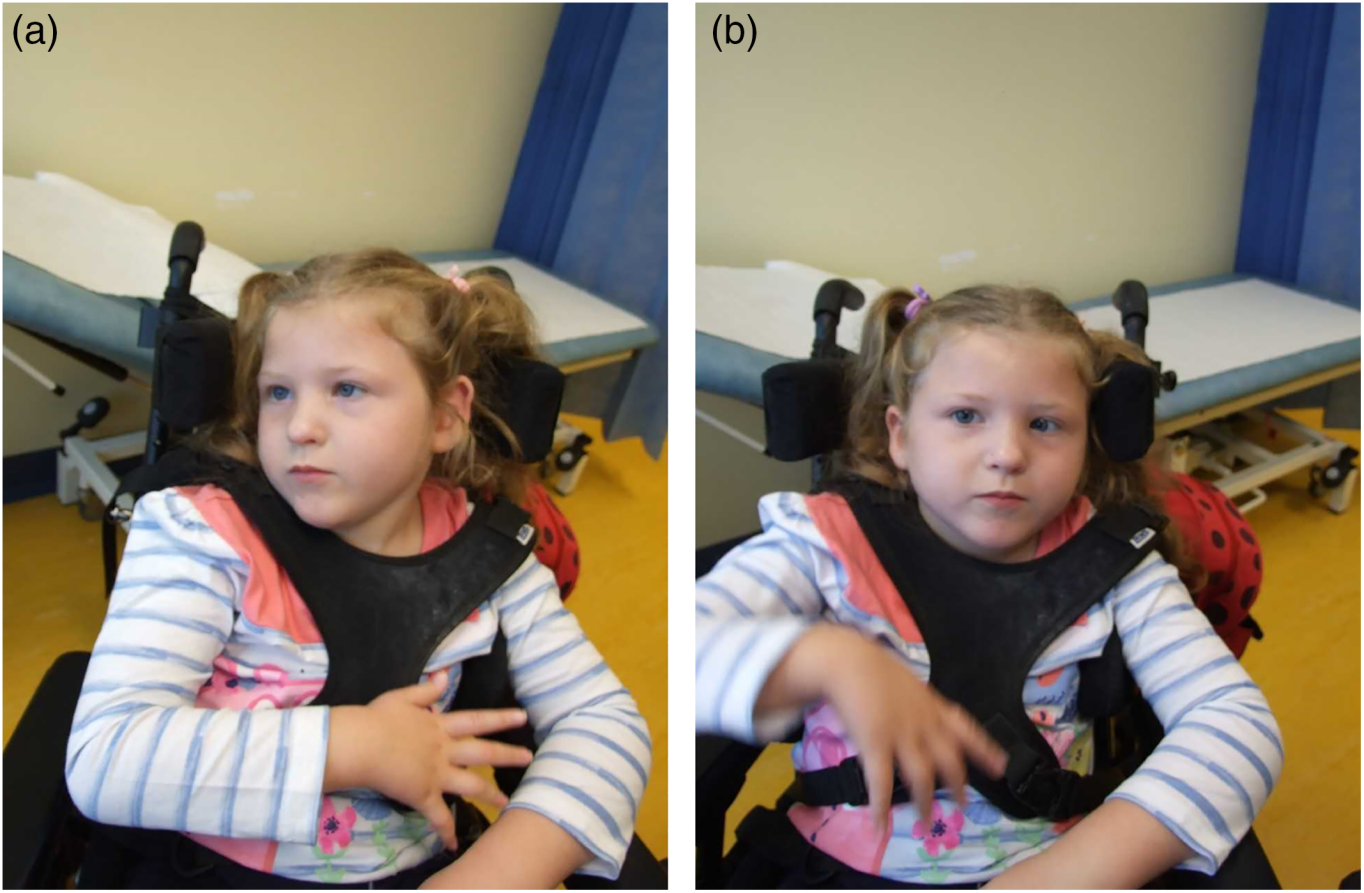
Child with biallelic CACNA1A variants, at age 9 years (published with parental permission)

The index child (female) was born at term via a normal vaginal delivery following an uneventful pregnancy. Head circumference and weight at birth were 25th–50th centile. She had hypotonia and weak suck. At 6 months, following the third routine scheduled immunizations, she developed low‐grade fever. Tonic–clonic status epilepticus ensued, resulting in admission to pediatric intensive care unit for ventilatory support and seizure management. Tonic–clonic seizures were refractory to multiple anti‐epileptic medications (levetiracetam, biotin, and pyridoxal phosphate). She was discharged on phenobarbitone and sodium valproate with nasogastric feeding. Over the next 3 years she required more than 20 admissions to hospital for chest infections, intractable seizures, and respiratory arrests requiring prolonged ventilatory support. She required a percutaneous gastrostomy due to poor bulbar function. Phenobarbitone, sodium valproate, and intermittent clobazam reduced seizure frequency. At age 3 years, phenytoin was introduced and phenobarbitone weaned off. At age 4 years, she was established on ketogenic diet and sodium valproate. At age 9 years, sodium valproate was withdrawn. She continues to have afebrile generalized tonic–clonic seizures, lasting for about 20–30 s, several times a week. These can cluster, sometimes in response to infection, resulting in status epilepticus which can be difficult to manage. Drooling is managed with glycopyrolate. On physical examination at age 3 years, she had secondary microcephaly (head circumference <0.4th centile). Occasional myoclonus was observed. There were no signs of spasticity. She showed anti‐gravity movements in all four limbs but no purposeful movement. Reflexes were difficult to elicit. At age 9, she had fixed flexion contractures in her elbows, hips, and knees. There were no distinctive dysmorphic features (Figure 1).

### 2.2 Developmental history and behavioral phenotype

At 5 months, she was diagnosed with an alternating convergent squint and delayed visual maturation. At 6 months (prior to epilepsy onset) she remained hypotonic with a weak cry. She smiled and made laughing noises, but did not make direct visual contact. At age 3 years, she remained profoundly hypotonic, and could not sit unsupported. She babbled, giggled, and smiled but not responsively. Developmental attainments and behavioral phenotype were assessed at age 10 years using standardized parent report measures. Vineland Adaptive Scales, 3rd edition (Sparrow et al., 2016) composite score was 26, corresponding to severe ID. Domain scores were Communication 20, Daily Living 30 (with a relative strength in the Personal sub‐domain), Socialization 24; motor skills 20. Gross motor age equivalent was 0 years 3 months; fine motor age equivalent was 0 years 0 months scores. The Developmental Behavior Checklist, 2nd edition (Einfeld & Tonge, 2002) did not highlight behavioral concerns (*T* scores: total 41, disruptive 38, self‐absorbed 44, communication disturbance 44, and anxiety 39) except for the social relating subscale (*T* score 53). The Social Responsiveness Scale, 2nd Edition (Constantino, 2012) highlighted impaired function across all social domains (*T* scores >60 for social awareness, social cognition, social communication, and social motivation). Restrictive and repetitive behaviors were not highlighted as an area of concern (*T* score < 60).

### 2.3 Family history

Parents are non‐consanguineous White British. No first or second degree relatives have been diagnosed with epilepsy, ataxia, motor impairment, or movement disorder. The child's father suffered from infrequent non‐severe migraines, which were not associated with motor symptoms or hemiplegia. Mild learning difficulties were suspected but not formally documented. The child's mother had no neurological symptoms or neurodevelopmental difficulties. The child's older brother had ametropia, speech and language delay, mild learning difficulties, and attention deficit hyperactivity disorder. The child's younger brother had mild learning difficulties.

### 2.4 Neuroimaging and neurophysiology

**FIGURE 2:**
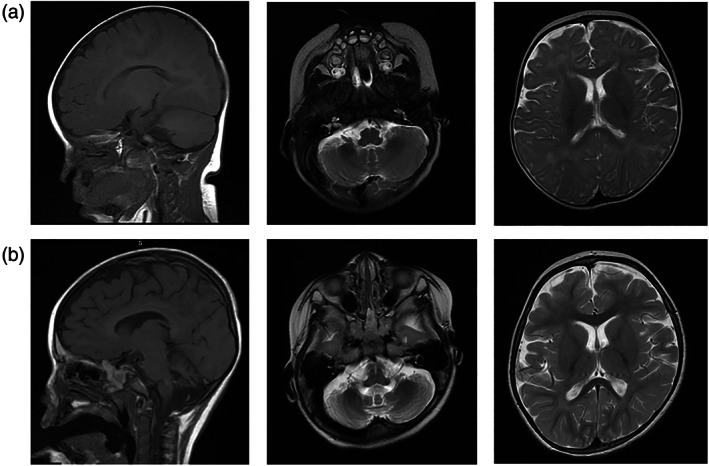
Magnetic resonance imaging for child with biallelic CACNA1A variants. (a) Cranial MRI at 6 months of age. (b) Cranial MRI at 3 years of age. Both panels show T1‐weighted sagittal image and T2‐weighted axial images

MRI at 6 months (Figure 2a) showed no cerebral malformation and age‐appropriate myelination, but mild cerebellar hypoplasia and thin corpus callosum. MRI at 3 years (Figure 2b) showed cerebellar volume loss involving both hemispheres, vermis and middle cerebellar peduncles. Ventricles and extra‐axial CSF spaces were mildly prominent, reflecting mild cerebral volume loss. CT head on two occasions provided no additional information. EEG during first admission to hospital (during morphine, phenytoin, and midazolam infusion) showed mild encephalopathy with intermittent isolated sharp waves over the posterior quadrants, and frequent tonic seizures. By age 8 years, recordings were dominated by continuous focal sharp and slow wave complexes over the posterior regions, plus brief tonic seizures which were not localized or lateralized. These EEG features are illustrated in Figure S1. Nerve conduction studies, electromyography, electroretinography, and audiology assessments were normal.

### 2.5 Diagnostic investigations

First line investigations for epileptic encephalopathy (infection screen and metabolic screen) were normal, including serum lactate (0.9 mmol/L), ammonia (50 μmol/L), blood spot carnitines, plasma amino acids, urine organic acid and amino acid, urine glycosaminoglycans and glycosaminoglycan‐typing, very long‐chain fatty acids, plasma pipecolic acid, serum biotinidase, serum TRF Isoforms, CSF lactate, CSF glucose, CSF protein, CSF Glycine, CSF Serine. Affymetrix GeneChip 6.0 SNP genotyping array identified a maternally inherited 516Kb duplication of 8q22.3, containing six genes; *CTHRC1* (MIM*610635), *SLC25A32* (MIM*610815), *WDSOF1*, *DCAF13*, *BX641143*, and *RIMS2* (MIM*606630). The population sampling probability of the duplication is >5% and dosage sensitivity score 1.04, indicating low likelihood of pathogenicity (https://www.deciphergenomics.org). 15q‐Methylation analysis for Angelman's syndrome and *SCN1A* sequencing were normal.

Next Generation Sequencing was carried out with targeted analysis of a 105 gene panel for epileptic encephalopathy, using the Illumina TruSight One sequencing panel. Two heterozygous sequence changes in *CACNA1A* were identified, and confirmed via Sanger sequencing. Parental testing for these variants via fluorescent sequencing showed that NM_001127221.2 c.2042_2043delAG, p.(Gln681ArgfsTer100) was maternally inherited, and NM_001127221.2 c.1693G>A, p.(Glu565Lys) was paternally inherited. The maternally inherited truncating variant has been previously reported as a pathogenic change associated with Episodic Ataxia 2, with variable expressivity within families (Denier et al., 1999; Kim et al., 2006; van den Maagdenberg et al., 2002). This is classified as a pathogenic variant by ACMG/AMP criteria (Richards et al., 2015). The paternally inherited missense variant has been reported twice in ClinVar as a variant of uncertain significance (www.ncbi.nlm.nih.gov/clinvar/variation/374438/). This variant has not been reported in The Genome Aggregation Database (gnomAD 2.2.1, https://gnomad.broadinstitute.org/) and therefore is considered very rare. It lies within a region demonstrating significant constraint for missense variants (observed 166, expected 437, missense constraint 0.38, *p*‐value 1.75 × 10 – 38). The variant is located within an ion transport domain. The missense change alters a highly conserved nucleotide and highly conserved amino acid (considering 11 species). In silico analyses classified this change as deleterious (SIFT, v6.2.0): deleterious (score: 0, median: 3.83); MutationTaster (v2013): disease causing (*p*‐value: 1). This variant remains of uncertain significance via ACMG/AMP classification: PM2_moderate (not in gnomAD), PP2_supportive (significantly constrained for missense variation, *Z* = 5.78) and PP3_supportive (conservative nucleotide and amino acid, in silico predictions indicated disease causing change).

## 3 DISCUSSION

Here, we describe a child with compound heterozygous *CACNA1A* variants, in context of previous reports of biallelic *CACNA1A* variants (Table S1). Consistent features which may aid variant interpretation and clinical management are infantile hypotonia, treatment‐refractory seizures with onset during first 6 months of life, cerebral visual impairment, profound developmental delay with prominent and persistent motor impairments, and progressive cerebellar changes on MRI. Collectively, these cases suggest a dose‐dependent effect of calcium channel dysfunction: monoallelic variants are associated with a highly variable spectrum ranging from asymptomatic to motor‐only syndromes to epilepsies and cognitive impairment; biallelic variants lead to severe and progressive neurological symptoms and cognitive disabilities. Two adult siblings reported by Lv et al. with biallelic truncating variants share features of refractory seizures, progressive cerebellar signs and cognitive decline, indicating potential for later age‐of‐onset.

This case illustrates the potential for a recessive diagnosis in an apparently sporadic disorder in which a de novo cause is initially considered more likely. It also highlights the significant challenges in variant interpretation and genetic counseling in biallelic cases. Biallelic variants could be identified by chance, leaving residual uncertainty about recurrence risk (either 25% or 50%, depending on true pathogenicity of both variants). Segregation studies are not always informative—parents with *CACNA1A* variants can manifest no symptoms or very mild and non‐specific features, which would not meet diagnostic criteria for any of the classical *CACNA1A*‐associated conditions. In this child, a known pathogenic truncating variant was associated with no symptoms in her mother, whilst an uncertain missense variant was associated with mild and non‐specific symptoms in her father not aiding interpretation of pathogenicity. Testing of siblings during childhood was not considered clinically indicated since identification of either variant would not have clear prognostic or treatment significance. Moreover, age‐related penetrance of phenotypes means that parental variants can be considered incidental findings with potential future health implications, raising significant anxieties.

There have been reports of successful treatment of seizures in individuals with monoallelic *CACNA1A* variants (Byers et al., 2016; Du et al., 2017). However, review of a larger case series of early onset cases suggests idiosyncratic responses to a wide variety of anti‐epileptic drugs, with a high proportion of cases remaining refractory (Le Roux et al., 2021). It is clear that new classes of mechanism‐informed anti‐epileptic drugs are needed to effectively manage seizures in individuals with monogenic DEE. Several experimental studies have suggested that calcium channel dysfunction impairs lysosomal fusion and autophagy (Zhu et al., 2021), which may explain the sensitivity of the cerebellum to progressive pathology in this disorder. Calcium channel activity is regulated by a large number of modifier proteins. Understanding how diverse pathogenic *CACNA1A* variants interact with these modifiers to mediate the highly variable expression of seizures and cognitive impairments could potentially uncover novel targets for disease‐modifying treatment.

## AUTHOR CONTRIBUTION

Kate Baker and Anna Maw conceived this research. Vivien W.Y. Wong‐Spracklen, Anna Kolesnik, Josefine Eck, and Saras Sabanathan carried out the literature review, collated clinical information, and collected behavioral phenotyping data. Olivera Spasic‐Boskovic carried out the genetic analysis. All authors wrote, revised, and approved the manuscript.

## CONFLICT OF INTEREST

The authors declare that there is no conflicts of interest.

## Supporting information


**Supplementary Figure S1** EEG characteristics of child with biallelic *CACNA1A* variants

**Supplementary Table S1** Genetic and phenotypic features of CACNA1A biallelic cases

## Data Availability

The data that support the findings of this study are available on request from the corresponding author. The data are not publicly available due to privacy or ethical restrictions.
