# Supplementary material for: Biallelic CACNA1A variants: Review of literature and report of a child with drug‐resistant epilepsy and developmental delay
Source: Am J Med Genet A. 2022 Sep 5;188(11):3306–11. doi: 10.1002/ajmg.a.62960 (PMC9826308; doi:10.1002/ajmg.a.62960)
Supplement: Supplementary file 1 — Supplementary Figure S1 EEG characteristics of child with biallelic CACNA1A variants [file AJMG-188-3306-s001.docx]

**Figure 1: EEG characteristics of child with biallelic *CACNA1A* variants**

1. **Age 7 months**

During initial hospital admission with intractable seizures, EEG is characterised by mild encephalopathy with intermittent isolated sharp waves over the posterior quadrants, and frequent tonic seizures.

**
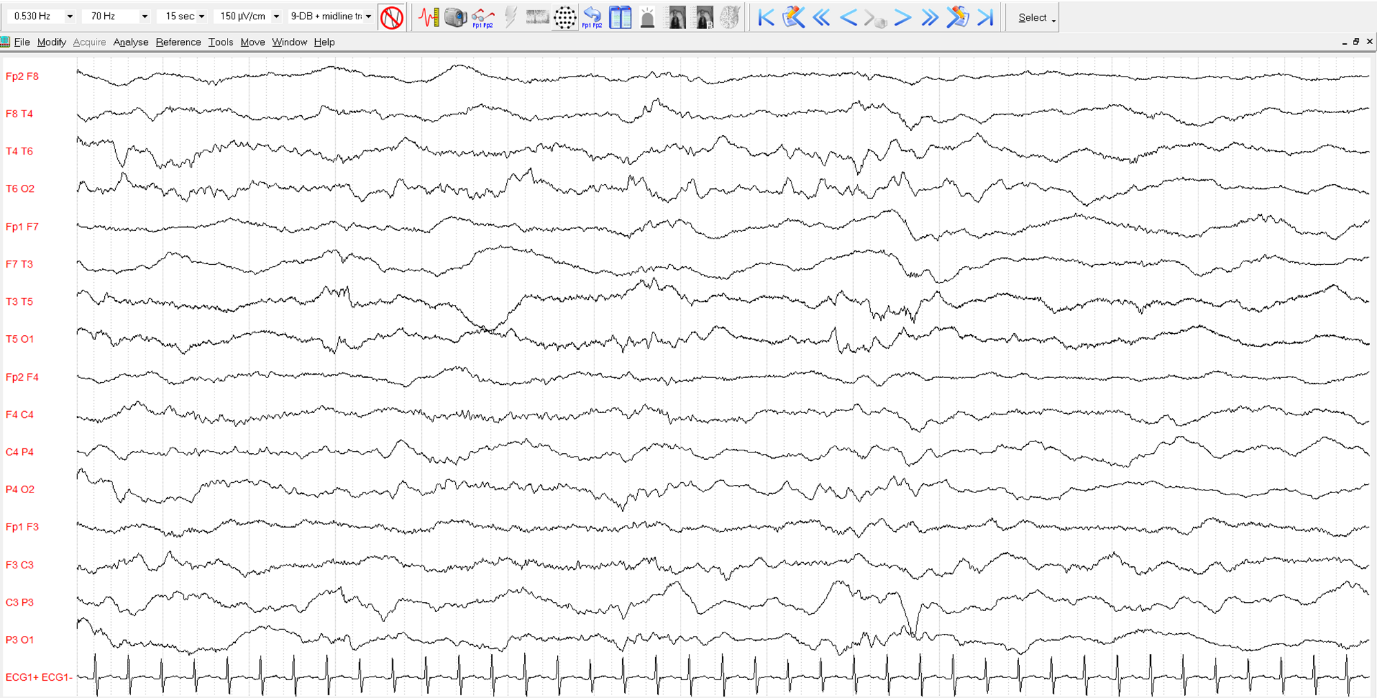
**

**
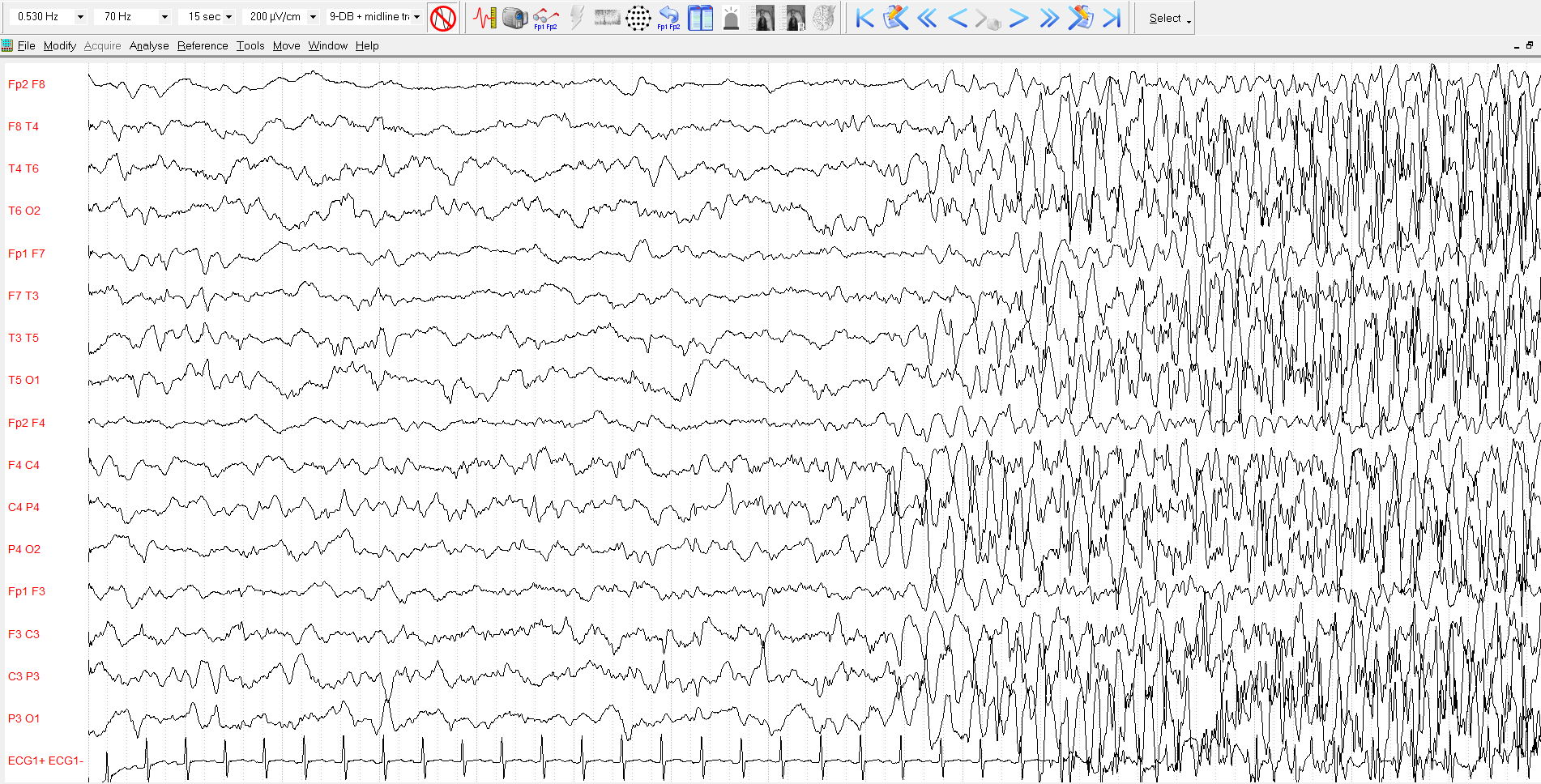
**

1. **Age 9 years**

EEG characterised by continuous focal sharp and slow wave complexes over the posterior regions, sometimes associated with altered awareness. Brief tonic seizures are observed, which are not localised or lateralised.

**
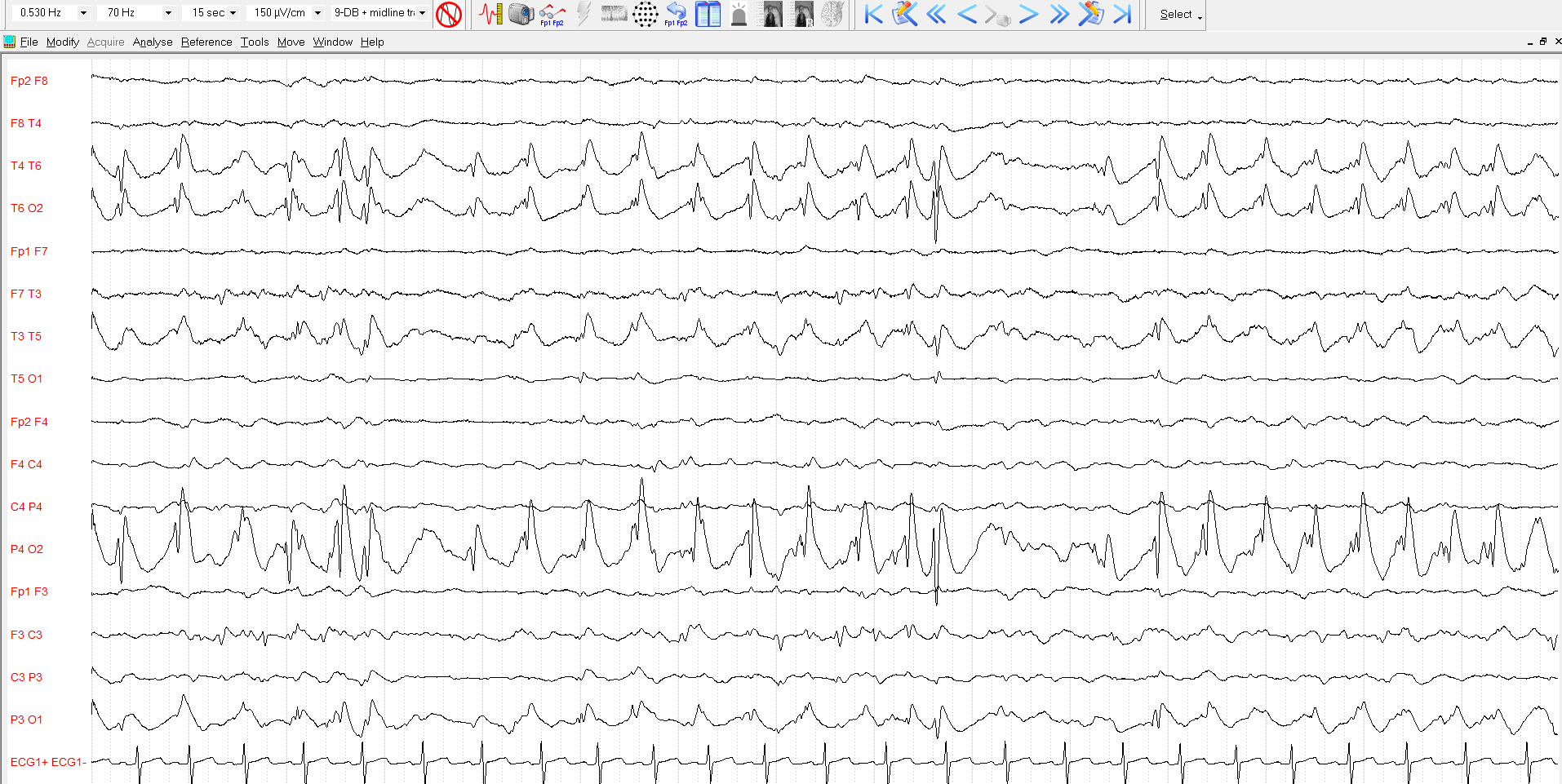
**

**
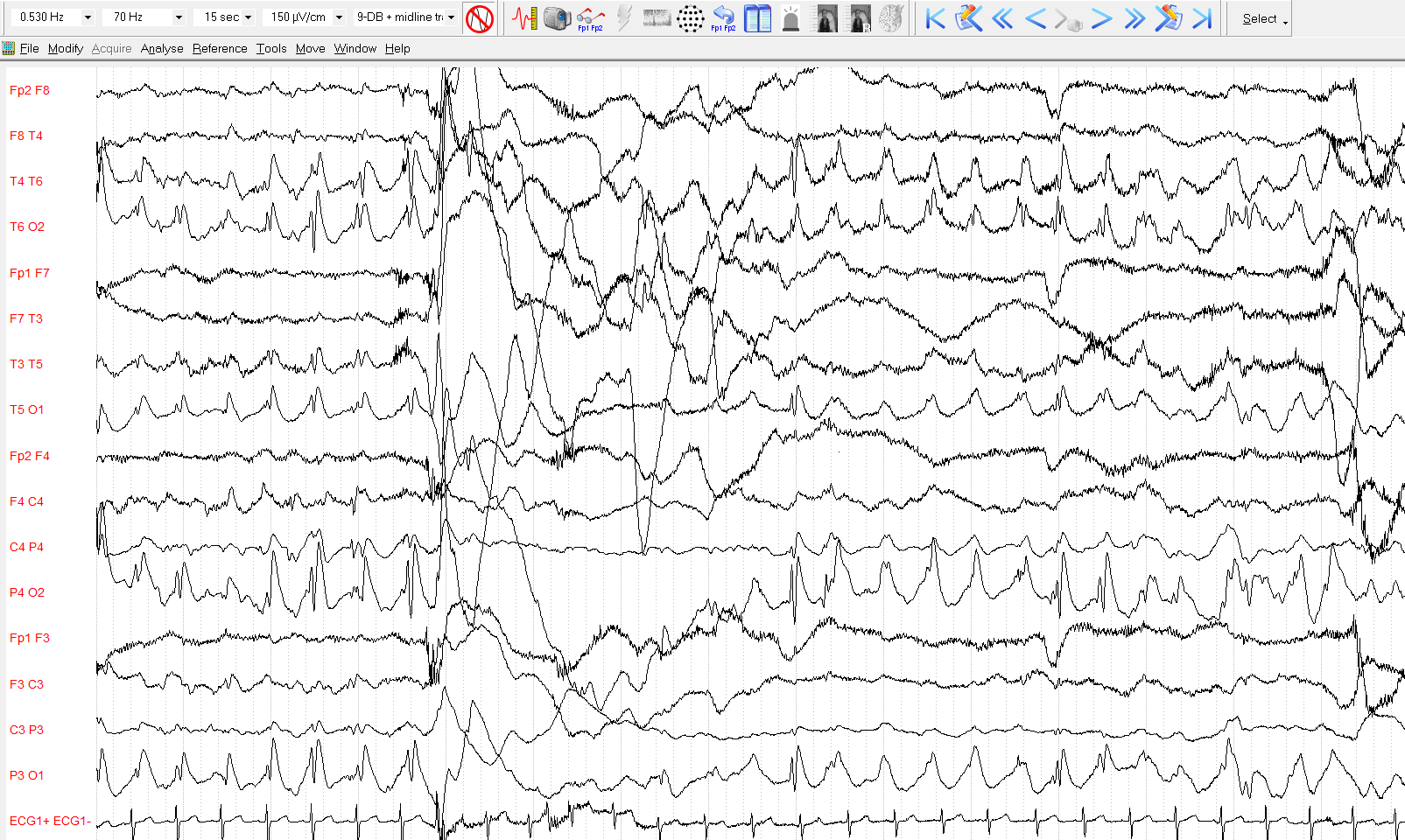
**
