# Supplementary material for: Biallelic CACNA1A variants: Review of literature and report of a child with drug‐resistant epilepsy and developmental delay
Source: Am J Med Genet A. 2022 Sep 5;188(11):3306–11. doi: 10.1002/ajmg.a.62960 (PMC9826308; doi:10.1002/ajmg.a.62960)
Supplement: Supplementary file 2 — Supplementary Table S1 Genetic and phenotypic features of CACNA1A biallelic cases [file AJMG-188-3306-s002.docx]

**Table 1: Genetic and phenotypic features of CACNA1A biallelic cases**

| **Publication** | **Current report** | **Reinson et al (2016)** | **Reinson et al (2016)** | **Arteche-López et al (2021)** | **Arteche-López et al (2021)** | **Arteche-López et al (2021)** | **Arteche-López et al (2021)** | **Lv et al**  **(2017)** | **Lv et al**  **(2017)** |
| --- | --- | --- | --- | --- | --- | --- | --- | --- | --- |
| **Case number** | I | I | II | I | II | III | IV | I | II |
| **Longevity / Age at last assessment** | 10 years | 5 years | died at 5 years | died within the ﬁrst 6 months of life | died within the ﬁrst 3 months of life | died within the ﬁrst 6 months of life | died within the ﬁrst 6 months of life | >50 years | >50 years |
| **Gender** | female | male | female | female | female | male | female | male | female |
| **Variant 1** | p.Gln681fs | p.Trp1439Arg | p.Trp1439Arg | p.Arg932* | p.Arg932* | p.Arg932* | p.Arg932* | p.A2326delinsGlnAla | p.A2326delinsGlnAla |
| **Variant 2** | p.Glu565Lys | p. Ala158Thrfs*6 | p. Ala158Thrfs*6 | p.Arg932* | p.Arg932* | p.Arg932* | p.Arg932* | p.A2326delinsGlnAla | p.A2326delinsGlnAla |
| **Pregnancy** | uneventful | polyhydramnios | n/a | uneventful | uneventful | uneventful | uneventful | n/a | n/a |
| **Birth** | SVD at term | uneventful | n/a | normal | normal | normal | normal | n/a | n/a |
| **Infancy** | Hypotonia | Hypotonia | hypotonia | Hypotonia | Hypotonia | Hypotonia | Hypotonia | n/a | n/a |
| **Epilepsy age-of onset** | 6 months | 4 months | 4 months | n/a | n/a | n/a | n/a | 50 years | 50 years |
| **Seizure types** | tonic-clonic, status epilepticus | tonic spasms | epileptic encephalopathy | epileptic encephalopathy | epileptic encephalopathy | epileptic encephalopathy | epileptic encephalopathy | progressive myoclonic epilepsy | progressive myoclonic epilepsy |
| **Epilepsy treatment** | partially responsive to phenobarbitone, sodium valproate, clobazam, ketogenic diet | n/a | n/a | n/a | n/a | n/a | n/a | refractory | refractory |
| **Motor milestones** | never sat independently, no fine motor skills | able to turn on his side | developmental age 1 month | n/a | n/a | n/a | n/a | normal | normal |
| **Communcation milestones** | no verbal communication | "arrested development" | developmental age 1 month | n/a | n/a | n/a | n/a | normal | normal |
| **Vision** | cerebral visual impairment | nystagmus, severe visual impairment | n/a | n/a | n/a | n/a | n/a | n/a | n/a |
| **ID and behavioural features** | severe ID, impaired social functions | n/a | n/a | n/a | n/a | n/a | n/a | cognitive decline | cognitive decline |
| **Neurological examination** | progressive contractures | At presentation: minimal spontaneous movement, absent tendon reflexes, positive Babinski; At 5 years: marked muscular atrophy and rigidity, Friedreich-like foot deformity | n/a | n/a | n/a | n/a | n/a | proximal and distal muscle atrophy of lower  limbs, muscle weakness, progressive ataxia | proximal and distal muscle atrophy of lower  limbs, muscle weakness, progressive ataxia |
| **Physical features** | secondary microcephaly | macrocephaly (+3SD), mild facial dysmorphology, intense salivation | n/a | mild facial dysmorphology | mild facial dysmorphology | mild facial dysmorphology | mild facial dysmorphology | n/a | n/a |
| **MRI** | at 6 months: mild reduction in cerebellar volume  at age 3 years: extensive cerebellar atrophy, mild cortical atrophy | small corpus callosum, dilatation of the frontal horns of the lateral ventricles,  diffuse hypomyelination, optic atrophy.  at 4 years: atrophic cerebellum and moderate cerebral atrophy | similar to sibling | n/a | n/a | n/a | n/a | normal | normal |
| **EEG** | irregular centrotemporal sharp wave activity, background slowing posterior maximal | multifocal sharp waves and spike-wave complexes; high voltage low amplitude background | multifocal interictal epileptiform discharges and ictal electric discharges. | n/a | n/a | n/a | n/a | generalized polyspike waves or generalized spike-slow complex wave | generalized polyspike waves or generalized spike-slow complex wave |
